# Containing COVID: the establishment and management of a COVID-19 ward in an adult psychiatric hospital

**DOI:** 10.1192/bjo.2020.126

**Published:** 2020-10-12

**Authors:** Melanie Knowles, Golnar Aref-Adib, Sarah Moslehi, Dominic Aubrey-Jones, Janet Obeney-Williams, Senem Leveson, Alina Galis, Alexandra Pitman

**Affiliations:** Camden and Islington NHS Foundation Trust, UK; Camden and Islington NHS Foundation Trust; and Division of Psychiatry, University College London, UK; Camden and Islington NHS Foundation Trust, UK; Camden and Islington NHS Foundation Trust, UK; Camden and Islington NHS Foundation Trust, UK; Camden and Islington NHS Foundation Trust, UK; Camden and Islington NHS Foundation Trust, UK; Camden and Islington NHS Foundation Trust; and Division of Psychiatry, University College London, UK

**Keywords:** In-patient treatment, COVID-19, physical health, staff well-being, psychiatry and law

## Abstract

**Background:**

As the coronavirus disease 2019 (COVID-19) epidemic in the UK emerged and escalated, clinicians working in mental health in-patient facilities faced unique medical, psychiatric and staffing challenges in managing and containing the impact of the virus and, in the context of legislation, enforcing social distancing.

**Aims:**

To describe (a) the steps taken by one mental health hospital to establish a COVID-19 isolation ward for adult psychiatric in-patients and (b) how staff addressed the challenges that emerged over the period March to June 2020.

**Method:**

A descriptive study detailing the processes involved in changing the role of the ward and the measures taken to address the various challenges that arose. Brief clinical cases of two patients are included for illustrative purposes.

**Results:**

We describe the achievements, lessons learned and outcomes of the process of repurposing a mental health triage ward into a COVID-19 isolation facility, including the impact on staff. Flexibility, rapid problem-solving and close teamwork were essential. Some of the changes made will be sustained on the ward in our primary role as a triage ward.

**Conclusions:**

Although the challenges faced were difficult, the legacy they have left is that of a range of improvements in patient care and the working environment.

On 11 March 2020, the novel coronavirus disease 2019 (COVID-19) outbreak was declared a global pandemic. Mental health in-patient facilities faced their own specific challenges in managing this crisis. One was the medical management of psychiatrically unwell in-patients who contracted COVID-19. There were two main components to this challenge: how to deal safely with deteriorations in physical health that did not meet the threshold for admission to an acute medical ward, and how to provide an enhanced level of physical healthcare that was within the team's skill range. We also needed to limit transmission in a setting where physical proximity between patients, and between staff and patients, can be much greater than in a general hospital, particularly in the context of agitated behaviour.

Camden and Islington NHS Foundation Trust is an acute mental health trust serving an ethnically diverse, inner-city population in North London, characterised by a high degree of income inequality and a high population turnover. As with other mental health hospitals, the majority of in-patients have leave (whether as informal patients or those under Section, subject to Section 17 leave) to go out on a regular basis, either alone or with staff, as well as frequent visitors. In contrast to a general medical setting, each ward has several communal spaces, and patients are encouraged to socialise and spend time in these areas. Many patients are admitted against their wishes under the Mental Health Act, and may be agitated, confused and lacking capacity to make basic decisions about their care and safety.

## Method

This is a descriptive study of (a) the steps taken by our mental health hospital to establish a COVID-19 isolation ward for adult psychiatric in-patients and (b) how staff addressed the challenges that emerged over the period March to June 2020. For illustrative purposes two patient's brief clinical cases are included.

As COVID-19 became formally acknowledged as a pandemic, and as government policy shifted to social distancing and then ‘lockdown’, the Trust made a number of decisions intended to limit transmission and safeguard patients and staff. On 13 March 2020 all carers groups and weekend support groups were cancelled, on Monday 16 March 2020 visiting was restricted to one family member per in-patient, and on 23 March 2020 the prime minister announced lockdown.

Over the same period cleaning schedules were intensified in all ward areas, community services moved to remote working, with care co-ordinators attending ward rounds remotely, and personal protective equipment (PPE) kit and barrier nursing training was rolled out across in-patient wards. The Trust's new ‘Health Based Place of Safety’, a recently-opened facility to assess and treat patients placed on Section 136 of the Mental Health Act, was used temporarily as an isolation area, providing up to five beds only, but it was clear that capacity needed to be created to provide a safe, isolated unit for in-patients with suspected and confirmed COVID-19. As the assessment ward of the Trust (i.e. the ‘port of entry’) it was felt by the medical and clinical directorate that our ward would be the most appropriate place for this. The ward manager and the two consultants, who both had a medical background (one in primary care and one in acute medicine) were consulted, and the decision was made to convert to a ward for COVID-19 patients from 18 March 2020. Our mental health trust was innovative in its approach to designating a COVID-19 isolation ward, implementing the change ahead of national guidelines.^1^ The aims were to isolate patients, contain the outbreak within the hospital, and focus on high-quality medical management of patients who became acutely physically unwell. Various challenges were apparent at the outset, and emerged over the course of this period, but the team worked together to find solutions, as outlined below.

Throughout the running of the ward, our practices were scrutinised by the various stakeholders – our own ethical standards as clinicians, our Mental Health Law experts, our ethics committee and our clinical and executive management.

### Beginning with the basics

The uncertainty we faced, and the rapidity of the changes that were made, cannot be overstated. Walking into the workplace and seeing colleagues dressed in surgical scrubs is something that in now commonplace in any general practice surgery or pharmacy, but at the time it was not something that any in-patient psychiatric facility had experienced in recent years. The sights and sounds of the ward changed overnight, and it is easy to forget now the profound and unsettling impact this had on both staff and patients.

Once the decision was taken to convert the ward, rapid decisions had to be made about day-to-day routine and structure. Some of the usual ward practice was adapted to make it more suitable for our revised role, with a daily ‘safety huddle’ of 10 min continuing and allowing staff to quickly air any concerns about the safety of patients or themselves. It became a means of creating a dialogue within and outside the ward, as this was also attended by the Trust infection control lead nurse and the Trust physical health lead nurse as well as initially a member of the senior management team. This was often where simple issues were identified and solved, for example the need to provide an individual observation monitor for each patient to avoid cross-contamination.

**Fig. 1:**
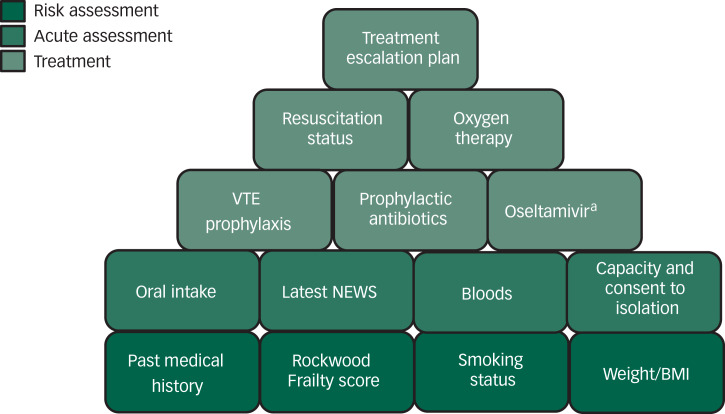
Elements included on the patient assessment proforma of the coronavirus disease 2019 (COVID-19) treatment plan. a. Initially included on the advice of the medical team at the acute trust, as it was part of their early treatment protocol, but this was removed after their guidelines were updated. BMI, body mass index; NEWS, National Early Warning Scores; VTE, venous thromboembolism.

A fixed and predictable daily routine was created to ensure some level of continuity for both staff and patients. We agreed early that the patients would be reviewed in person daily by the doctors, representing an increase relative to the usual practice of patients being seen as needed, as well as at least once weekly consultant reviews. We also developed a detailed patient assessment proforma focusing on the COVID-specific issues (see Fig. 1 for the elements included on the proforma).

Access to PPE was a widespread concern nationally in the early days of the pandemic. National guidelines changed on a frequent basis.^2^ Access to gloves, aprons and surgical masks was essential from the start, and Trust personnel responsible for procuring these worked alongside us to ensure that our PPE met the need for the clinical tasks we were performing.

**Fig. 2:**
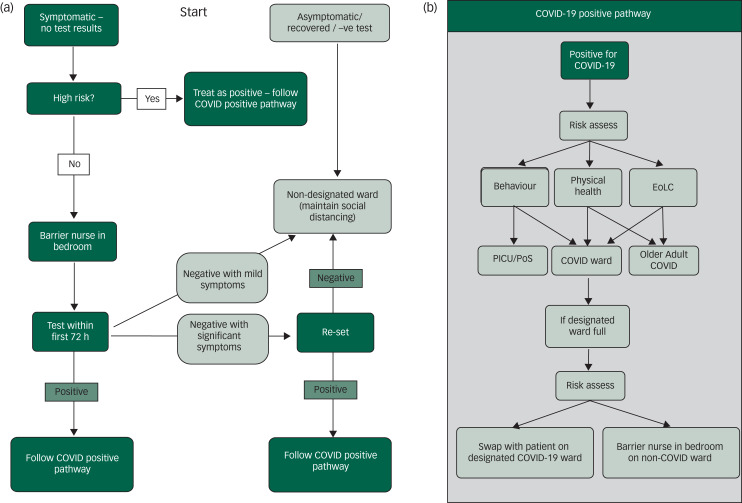
(a) Flow chart for patients who are symptomatic, asymptomatic or negative for COVID-19. (b) COVID-19 positive pathway. EoLC, end of life care; PICU, psychiatric intensive care unit; PoS, place of safety.

Early discussions focussed on what exactly the ward's role would be in relation to our local acute hospitals, and how far the medical management of unwell patients would be taken. We quickly arrived at the consensus that our role was to isolate, stabilise and medically manage these patients. Before testing began, patients were moved to our ward if they had suspected COVID-19 status and if, after 7 days in isolation (i.e. to avoid infection by other patients on our ward), they were asymptomatic then they were stepped back to their original psychiatric ward (Fig. 2(a)).

In the early stages of the epidemic nationally we faced the very real possibility that local medical wards and intensive care units would become overwhelmed, and that we would take on responsibility for managing patients with a severity of physical illness that was beyond our usual remit and capacity. We discussed anticipated palliative care needs and whether we should consider acquiring piped oxygen. Some limits were immediately apparent: psychiatric nurses are not trained in the management of intravenous medication and it would not be safe to nurse patients requiring parenteral administration. However, others issues were less clear, necessitating ongoing discussion. For example, in preparing to deliver palliative care, we ensured that supplies of morphine were adequate and refreshed local protocols appropriate to our skill levels.^3^

### Keep talking: working with our medical colleagues

At the onset of the epidemic there were clear challenges in dealing with a novel virus with many still unknown symptoms and unpredictable complications. We enhanced our existing close ties with medical teams at the local acute hospital, who usually conduct regular outreach clinics in our hospital and were familiar with the potential challenges in caring for our acutely unwell psychiatric population.

Regular virtual conferences were held with senior medical and nursing colleagues from the acute Trust, to discuss the implications of new information as it emerged daily. One example was how to manage the elevated mortality risks in patients infected by COVID-19 who are smokers, have renal or cardiovascular risk factors, or belong to Black and minority ethnic groups.^4^ A respiratory nurse specialist delivered training on the ward in oxygen prescribing and administration, and helped us clarify the threshold of oxygen treatment we would provide on our ward. This allowed us to ensure safe and adequate oxygen supplies, and prepare new resources such as oxygen concentrators not generally used in psychiatric wards. Discussions about respiratory distress rarely arose on psychiatric wards pre-COVID, but we used the staff safety huddle as a forum. This safety huddle was regarded as an essential part of our routine in highlighting every aspect of risk, as well as providing opportunities to reflect as a group and share feelings.

When our patients decompensated physically, the established dialogue between our ward and the acute medical wards was an invaluable way of accessing specialist advice rapidly. Some patients moved relatively frequently between our ward and the medical ward and back again. Thresholds for transfer were arrived at collaboratively, facilitating agreements about transfer much more smoothly. The relative ease of agreeing such transfers reflected a mutual recognition that individual circumstances could change quickly in relation to a patient's severity.

### Unwell in body and mind

There were three main groups of patients who were admitted to our COVID-19 isolation ward:

(a) those with very mild COVID-19 symptoms who were settled and able to self-isolate without any major difficulty;
(b) those with severe COVID-19 symptoms who required intensive medical care; and
(c) those who were agitated and could not agree to self-isolate.

The first group with mild symptoms were monitored by nurses each day, with a minimum four times daily National Early Warning Scores (NEWS) performed, and ongoing interaction with staff on a relational level. We were aware of the frustrations of being isolated within one room, particularly with no visitors, and the risk of this disconnectedness in an unfamiliar place contributing to a deterioration in mental state. The occupational therapists worked hard to enrich the environment with activity packs, radios and iPads. We obtained written feedback throughout this period from patients who were completing their isolation period. They provided feedback about how disconnected they felt from the outside world, suggesting that we should help them gain access to regular video contact with friends and family, which we did via the Attend Anywhere secure video service. This medium was also used to allow remote attendance of relatives to medical reviews and meetings, and the patients and relatives fed back that they felt much less anxious and uncertain as a result.

The second group with severe COVID-19 symptoms and who had clearly deteriorated physically were managed with enhanced care, including one-to-one nursing and oxygen up to a maximum concentration of 4 L/min. Parallel adjustments were made in the two older adult wards within our mental health (of ten wards in total) unit to accommodate the patients with COVID-19 whose nursing needs were more appropriately met in a setting with older adults nursing expertise (Fig. 2(b)). Key to the management of medically unwell patients was the rapid development of a new COVID-19 proforma (see Fig. 1 for the elements included on the proforma) for medical reviews, which was updated whenever new Trust or national guidelines were produced. This included space to record resuscitation status, indications for venous thromboembolism prophylaxis and oxygen, nutritional status of the patient, and whether antibiotics were indicated.^5^ The proforma also flagged up potential risk factors such as high (or very low) body mass index, frailty, smoking status and the presence of diabetes and respiratory comorbidities.

Challenges for the third group of patients, who were agitated and unable to self-isolate, included the need to confine them in a small room with one-to-one nursing, yet fewer internal resources to handle these challenges in the context of their acute mental illness. Patients’ capacity to consent to isolation was assessed and mental health legislation interpreted to accommodate the unprecedented context of a global pandemic. In consultation with our restrictive practice lead and mental health law hub, we developed a pathway whereby the isolation of patients, whose refusal was linked to their mental disorder and/or who lacked capacity to consent to self-isolation, could be enforced under the Mental Health Act or Mental Capacity Act, to safely protect both our patients and our staff from the risk of transmission and to ensure patients complied with government guidance. The relevant legal framework was determined on a case-by-case basis, with reference to our ethics committee and support from our legal advisers as required. Statutory powers were used with full regard to the principles of the Code of Practice to the Mental Health Act and of the Mental Capacity Act, which involved 1∶1 nursing observations and frequent medical reviews. This allowed us to keep the use of restriction proportionate to the risks involved and facilitate early termination when possible.

Providing access to iPads and facilitating virtual contact with friends and family was essential in mitigating some of the distress. The nursing team engaged them in conversation and activities when possible. While we attempted at all times to manage patients with empathy and in a relational way, sedation was sometimes indicated for the most agitated patients, and this was calibrated and administered carefully. Patients whose symptoms and behaviour could not be managed on our ward were transferred to our psychiatric intensive care unit (Fig. 2(b)), as per usual protocols, where the more secure facilities and nursing training were more appropriate to these patients’ needs.

#### Case illustration of a patient with severe COVID-19 symptoms

A 50-year-old woman, who was a non-smoker with type 2 diabetes and obesity, had been admitted to a mental health treatment ward because of a relapse in psychotic symptoms in the context of a severe and enduring mental illness. Over the next 3weeks she became intermittently severely physically unwell with frequent episodes of oxygen desaturation, pyrexia and tachycardia. She was transferred to our ward with suspected COVID-19, and was transferred several times from our Trust to the neighbouring acute medical Trust and back depending on her level of need, which changed frequently. The challenges included maintaining a safe nutritional status, being able to respond quickly to sudden deterioration and respiratory distress, and maintaining her morale in the context of both her psychotic and physiological symptoms. The physical impact of a long hospital stay, including muscle wastage and deterioration in mobility, was addressed with physiotherapy on our ward. Her mental state improved on treatment with antipsychotic medication and mood stabilisers. After 3 weeks, she was ready for discharge to her home. Staff applauded when she walked off the unit; almost everyone had seen her at her most unwell and had been aware of her potentially poor prognostic factors.

#### Case illustration of a patient who was agitated and unable to self-isolate

A man in his twenties had been admitted to our mental health unit some months earlier and was under Section 3 of the Mental Health Act with a diagnosis of a first psychotic episode. He was transferred to our COVID-19 ward when he presented with a new-onset dry cough prior to testing becoming available. His NEWS score remained below 2 at all times. However, he was agitated and confused, and lacked capacity to consent to self-isolation. At times he became distressed and verbally angry. He was nursed under close observation (one-to-one) and provided with an iPad and tailored occupational therapy activities to engage him. He was supported in calling his parents regularly, and this was incorporated into his treatment plan as the first line in managing his distress. When he left his room, as he frequently did, the team was able to verbally redirect him to return without the need for physical restraint. He was transferred back to his treatment ward 7 days after his symptoms had started, to continue his assessment and treatment, and no major incidents occurred during his in-patient stay with us.

## What about the staff?

As with many work places, the need for some staff members to isolate or shield because of their risk of acquiring COVID-19, a household member's vulnerability, or because they or a household member had developed symptoms, meant that a new team was formed from staff based on other wards across the Trust. Community nurses also joined the team, some of whom had enhanced physical health skills. The new configuration of our team afforded both opportunities and challenges. As a new team we worked together to agree on the new processes required, and there were opportunities for staff to learn skills such as phlebotomy or more advanced assessment of respiratory function.

The number and rapidity of changes had an impact on staff stress levels, all of whom were required to adapt quickly to working in a new environment in which they faced threats to their physical health. One member of the nursing team commented that they felt ‘out of my depth, and scared of what it would be like caring for someone who was very unwell’. Another recalled ‘just how long everything took – giving medication to patients when we had to change in and out of PPE each time took forever’. All members of the team adopted a high level of flexibility when tasks were allocated; essentially ‘mucking in’ as required. When our domestic services faced challenges of staff absence, the multidisciplinary team helped by cleaning and disinfecting the ward, regardless of seniority.

A clinical psychologist visited the ward regularly to address patient and staff well-being. The existing ward structure of staff reflective practice was increased to weekly meetings with voluntary attendance, although meetings were well attended by all available staff on shift. This provided opportunities to reflect on learning and challenges of the rapidly changing work environment and safe space for staff to share their experience and foster mutual support and cohesion in a newly formed team.^6,7^ There are widespread concerns, based on evidence from previous epidemics, that the COVID-19 pandemic will increase the risk of distress and trauma symptoms for front-line healthcare staff.^8^ These meetings provided an opportunity to normalise and validate psychological reactions to stress when raised by staff and the promotion of individuals’ coping strategies and resilience^9^ alongside providing information about the Trust well-being support options for staff.

## Test, test, test

**Fig. 3:**
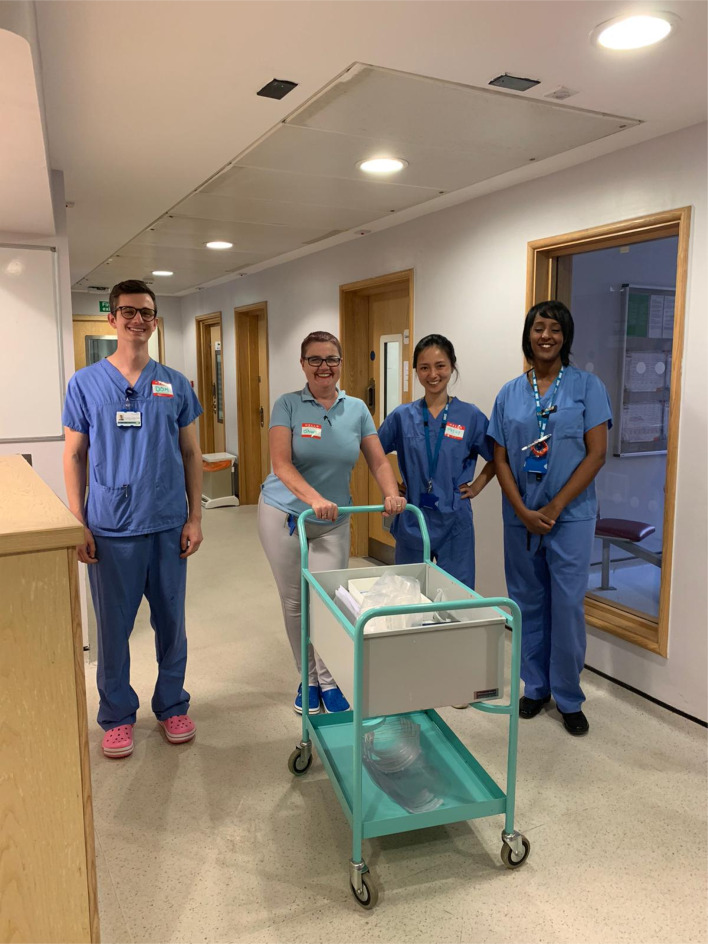
Members of the Sapphire Team conducting testing and training on other wards.

COVID-19 swab testing became available to patients on the COVID-19 ward on 26 March 2020, which enabled the prevalence of the virus to be established. The physical health lead nurse trained six members of the COVID-19 clinical team to conduct the tests, and they in turn provided outreach training in this to the other wards (Fig 3). The skill was easily acquired and facilitated rapid identification of new cases. From 26 March, we changed our admission protocol such that we were only caring for patients with a confirmed positive COVID-19 status. This was to avoid the risk of untested patients contracting the virus. As testing became more widely available, we were able to screen every patient at the point of admission to any ward on our Trust's in-patient units, and identify symptomless carriers of the virus. These individuals were then transferred to the COIVD-19 ward for isolation.

## Discussion

### Legacy

Some of the changes we made during this pandemic period will be sustained on the ward in our primary role as a triage ward, particularly as we anticipate a possible second wave of COVID-19 and subsequent winter resurgences. The ward has not been ‘stepped down’ entirely to its previous role. We have furloughed beds in an area of the ward that can be isolated and we will be able to respond within 24 h of any potential upsurge.

At the suggestion of medical and nursing staff members, the daily routine has been altered to accommodate a more inclusive morning safety huddle and handover, ensuring that as many staff as possible are present for this. We are adopting a more flexible and patient-centred approach to all psychiatric reviews and meetings by allowing patients more autonomy over when and where they are seen. We have abolished formal ‘ward rounds’, with a junior doctor typing notes during the meeting, which patients reported finding quite stressful, and have doubled the amount of time allocated to accommodate a patient review. New members of the nursing team continue to be trained in phlebotomy and we are piloting a more intensive physical health screening system as part of our daily practice. Although the challenges we faced over this period have been difficult, the legacy they have left is that of a range of improvements for patient care and working environment.

### Implications

Our experiences in repurposing a mental health triage ward into a COVID-19 isolation facility for psychiatric patients demonstrated the need for flexibility and rapid problem-solving abilities in all the staff members on the team. Continual dialogue between ward staff and senior management and other specialist staff members was essential, as was the ability to adapt our processes continually as information and protocols evolved locally and nationally.

Our experiences have provided a valuable opportunity to consider how we might revisit this service model in the event of a second wave. In reflecting upon both the successes and potential pitfalls of this initiative we feel that key factors contributing to the safe and efficient management of the ward were our frequent discussions with the medical team in the neighbouring Trust, the recruitment of community matrons with specialist physical health knowledge, the timely procurement responses of senior management to the changing role of the ward and the prioritisation of the morning safety huddle, which allowed staff to air concerns and solve problems collaboratively. Another critical factor was gaining collective agreement at an early stage as to the limits of care we could provide safely on a psychiatric ward, ensuring that if indicated, patients were transferred to a medical ward without delay.

The COVID-19 crisis has been described by senior officials as ‘unlike anything ever seen in peacetime’. We have needed to respond with enhanced degrees of flexibility, creativity and resilience as new intelligence has emerged and, like all post-war environments before us, we are ‘rebuilding back better’.

## Data Availability

Data are not available for reasons of patient confidentiality.
